# Shallow tillage mitigates plant competition by increasing diversity and altering plant community assembly process

**DOI:** 10.3389/fpls.2024.1409493

**Published:** 2024-08-07

**Authors:** Zihao Li, Jirong Qiao, Zhuofan Li, Xiaowei Gao, Guangyu Hong, Haifeng Yang, Ercha Hu, Chenming Liu, Xiaojiang Wang, Guanzhi Liu, Runhong Gao

**Affiliations:** ^1^ Institute of Forest Ecology, Inner Mongolia Academy of Forestry Sciences, Hohhot, China; ^2^ College of Forestry, Inner Mongolia Agricultural University, Hohhot, China; ^3^ College of Grassland, Resources and Environment, Inner Mongolia Agricultural University, Hohhot, China

**Keywords:** shallow tillage, species diversity, phylogenetic structure, community assembly, Mu Us Desert

## Abstract

**Introduction:**

Understanding how human activities affect biodiversity is needed to inform systemic policies and targets for achieving sustainable development goals. Shallow tillage to remove *Artemisia ordosica* is commonly conducted in the Mu Us Desert. However, the impacts of shallow tillage on plant community species diversity, phylogenetic structure, and community assembly processes remain poorly understood.

**Methods:**

This study explores the effects of shallow tillage on species diversity including three a-diversity and two b-diversity indicators, as well as phylogenetic structure [phylogenetic diversity (PD), net relatedness index (NRI), and nearest taxon index (NTI)]. Additionally, this research analyzes the effects of shallow tillage on the community assembly process.

**Results and discussion:**

The results showed that the a-diversity index, b-diversity index, and PD of the shallow tillage (ST) communities were significantly higher than those of the non-shallow tillage (NT) communities, and the phylogenetic structures of both the ST and NT communities tended to be differentiated, with competitive exclusion being the main mechanism of plant assembly. However, shallow tillage increased the relative importance of the stochastic processes dominated by dispersal limitation, mitigating plant competition in the communities. This conclusion was supported by the Raup–Crick difference index-based analysis.

**Conclusion:**

Therefore, for the ecological restoration of the Mu Us Desert, species with adaptability and low niche overlap should be selected to increase the utilization efficiency of the environmental resources. The results of this study provide a foundation for policy development for ecosystem management and restoration in the Mu Us Desert.

## 1 Introduction

Biodiversity underpins ecosystem stability and social development, yet it is declining globally due to human activities (Zhang and Chen, 2022; Zhang et al., 2023). Desert ecosystems cover 12% of the Earth’s land surface (Durant et al., 2012; Chen et al., 2023) and, compared to other ecosystems, are environmentally fragile and sensitive to disturbance, which can have disproportionately severe impacts on biodiversity. Previous studies have shown that even the smallest soil disturbance can significantly alter plant community structure and biodiversity in arid ecosystems (Valone, 2003; Erfanian et al., 2019). Shallow, a common disturbance measure in agroecosystems, is also used for desert grassland improvement, whereby local people remove vegetation that does not provide economic benefits by plowing it to utilize desert grassland resources. This practice, although tailored to local needs, significantly risks community species composition biodiversity and the natural processes supporting desert ecosystems (Randriamalala et al., 2012; Cordeau et al., 2017). Consequently, understanding the ecological consequences of anthropogenic interventions is essential for developing more sustainable management practices (Jaureguiberry et al., 2022).

Community assembly is an intrinsic mechanism of biodiversity change and maintenance and can be used to assess the impacts of external drivers, such as human activities, on biodiversity (Chase, 2010). Ecological niche theory (based on deterministic processes) and neutral theory (based on stochastic processes) are the two hypotheses for the community assembly process, and it is widely accepted that deterministic and stochastic factors work together in the community assembly process. However, assessing the relative importance of these factors is challenging, especially in arid regions. Traditionally, species α-diversity and β-diversity are often used in community assembly process analyses (Mori et al., 2018; Jin et al., 2023). However, species diversity reflects only one dimension of biodiversity and does not consider evolutionary diversity (Capmourteres and Anand, 2016; Heino and Tolonen, 2017). Therefore, the use of phylogenetic structures to analyze community assembly processes has gained prominence (Cadotte et al., 2009; Mishler et al., 2014). This methodology not only assesses the evolution of biodiversity and evolutionary distances among taxa but also reflects functional diversity, as closely related species often exhibit similar traits (Cadotte et al., 2008). Although species diversity and phylogenetic structure are often highly correlated, they may indicate different patterns of community assembly (Tucker and Cadotte, 2013). Accordingly, it is vital to integrate various measures of biodiversity to achieve a more comprehensive understanding of community assembly. Uncovering these hidden patterns can provide more targeted guidance for restoring damaged ecosystems and deepen the understanding of diversity formation and maintenance.

The studies on the effects of tillage on community diversity and assembly processes have mainly focused on agroecosystems (Travlos et al., 2018). It has been shown that the removal of native vegetation by tillage creates a large number of gap ecological niches that favor plant species colonization (Czerwiński et al., 2018). Consequently, tillage alters the species composition of weed communities, in which perennial species shifted to annual species, and increases the community evenness index (Booth and Swanton, 2002) but does not change diversity indices such as species richness and the Shannon–Weiner index (Légère et al., 2005; Randriamalala et al., 2012; Barroso et al., 2015). Tillage imposed environmental filtering, an artificial deterministic process, on the weed community and therefore altered the drivers of weed community assembly (Alarcón et al., 2018). However, the relative importance of deterministic and stochastic processes is largely influenced by community type (Cordeau et al., 2017). Although phylogenetic structure is important for understanding the mechanisms of community diversity change and formation, little is known about the effects of tillage on plant phylogenetic structure.

*Artemisia ordosica* plays a crucial role in wind and sand control (Wei et al., 2016). However, its strong odor leads to its unpopularity among livestock. Consequently, local herders frequently remove it through shallow tillage, which is defined as soil disturbance up to a depth of 10–15 cm (Cooper et al., 2016; Laudicina et al., 2017) to improve grasslands. This practice promotes the growth of more palatable herbaceous plants to support production and livelihoods in the Mu Us Desert but also heightens the risk of desertification (Wang et al., 2017). However, less research has been conducted on shallow tillage on plant diversity and community assembly processes in desert grasslands. This study evaluates the short-term impacts of shallow tillage on plant communities in the Mu Us Desert, focusing on changes in species diversity and phylogenetic structure, as well as community assembly processes, before and after tillage. Specifically, we aim to answer the following: (1) “Does shallow tillage affect the species diversity and phylogenetic structure of the communities and how?” and (2) “Does shallow tillage affect the ecological processes that drive community assembly?” We propose the hypothesis that shallow tillage increases community species and phylogenetic diversity but does not alter its community assembly processes because studies have shown that shallow tillage leads to a large number of blank ecological niches, while multiple tillage alters weed community assembly processes. The study of the effects of shallow tillage on multilevel of biodiversity (species diversity and phylogenetic diversity) of desert ecosystems is an important reference for conservation strategies and land management practices.

## 2 Materials and methods

### 2.1 Study area

**Figure 1:**
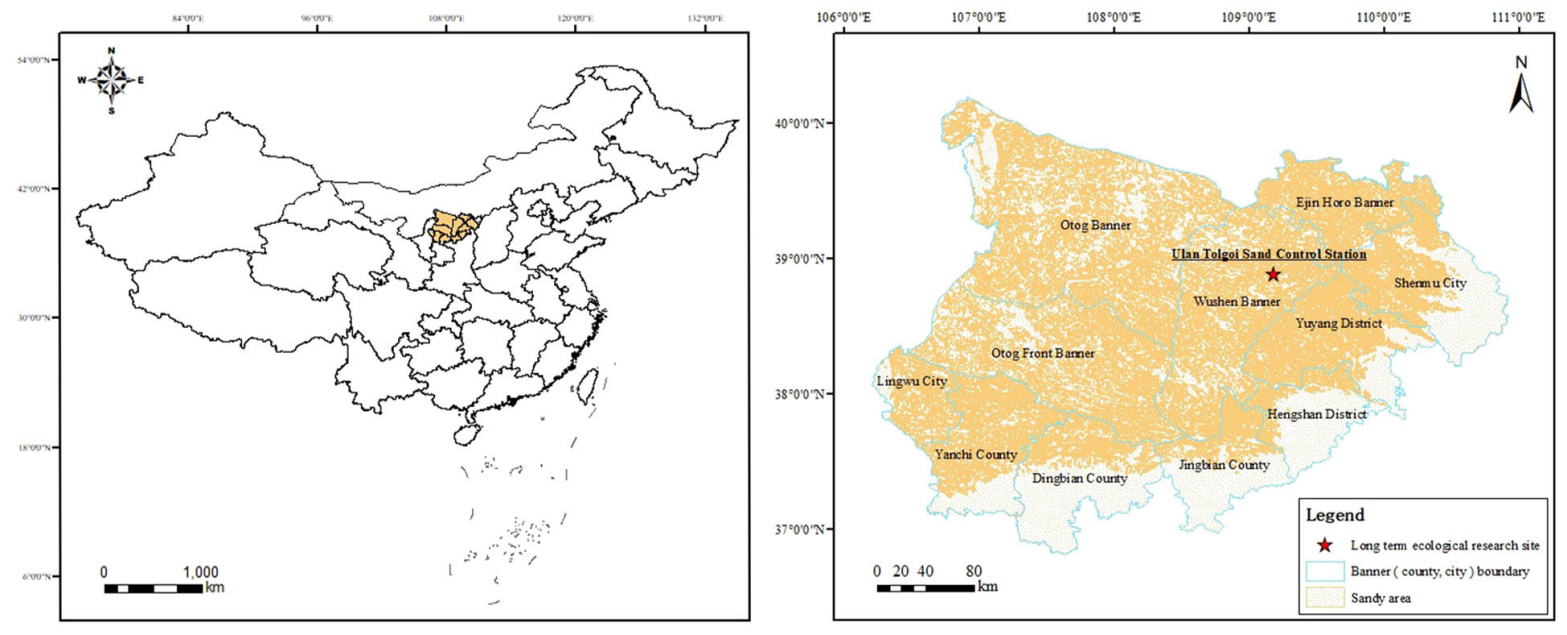
Geographic location of the study area.

The study was conducted in a fixed monitoring site at the Ulan Tolgoi Desertification Control Station located in Wushen Banner (38°48′N, 109°19′E, at 1,390 m), Inner Mongolia, China (**Figure 1**). This region in the hinterland of the Mu Us desert has a semi-arid continental monsoon climate, which is dry and windy. The average annual temperature ranges from 6.0°C to 9.0°C (Zheng et al., 2019a). The annual precipitation in the area varies greatly, ranging from 250 to 400 mm, and the precipitation is mainly concentrated in July–September, accounting for up to 75% of the annual precipitation. The precipitation in August can account for up to 54% of the whole rainy season, and the average annual evaporation ranges from 2,100 to 2,600 mm (She et al., 2017). The predominant soil type is sandy (FAO soil classification), and the geomorphological types include fixed, semi-fixed, wandering dune, and inter-dune lowlands (Chen et al., 2022). Drought-tolerant plants, such as *A. ordosica*, *Salix cheilophila*, *Caragana microphylla*, and *Corethrodendron fruticosum* var. *mongolicum*, are widely distributed in the area (Zhang et al., 2018).

### 2.2 Sample plots and data collection

In March 2022, shallow tillage was applied to the *A. ordosica* community (there is no history of any tillage). In mid-August of the same year, 10 shallow tillage (ST) strips and 10 non-shallow tillage (NT) strips, each approximately 8 m wide and 200 m long, were selected, in which the two were interleaved, and the distance between adjacent strips under the same treatment was greater than 200 m to include more heterogeneous habitats. Three 8 m × 20 m sample plots were randomly set up in each shallow-tillage and non-shallow-tillage strip, and a detailed survey of plant species and their abundance occurring in the plots was conducted (**Supplementary Table S1**) to assess species diversity and phylogenetic structure.

### 2.3 Species diversity and phylogenetic structure

#### 2.3.1 α-Diversity

Based on the species matrix data that were obtained from the survey, we calculated the Shannon–Weiner index (H), Simpson’s index (D), Pielou’s evenness index (J), and the cumulative number of species as measures of species α-diversity, and the formulas are as follows:

Shannon diversity index:


$$H=-\sum_{i=1}^{S}P_{i}\ln P_{i}$$

Simpson’s index:


$$D=1-\sum_{i=1}^{S}P_{i}^{\;2}$$

Pielou’s evenness index:


$$J=\frac{H}{\ln S}$$

where S is the number of species and P_i_ is the relative abundance of species *i*. P_i_ = n_i_/N, where n_i_ represents the number of individuals of species *i* and N represents the total number of individuals of all the plant species in a particular sample.

#### 2.3.2 β-diversity

The Bray–Curtis dissimilarity index (β_BC_) and the Jaccard dissimilarity index (β_J_) were used to explore the β-diversity characteristics of the plant communities in the shallow and non-shallow tillage belts. The β_BC_ is based on the species multiplicity matrix to measure the compositional dissimilarity between the plots based on differences in species abundance, and it ranges from 0 (when both plots have the same species with equal abundances) to 1 (when the plots do not share any species; Catalán et al., 2023). The β_BC_ was calculated using the following equation:


$$\beta_{BC}=\frac{\sum_{i=1}^{S}\left|x_{ij}-x_{ik}\right|}{\sum_{i=1}^{S}x_{ij}+x_{ik}}$$

where S is the number of species in the sample-species matrix, and x_ij_ and x_ik_ are the abundance of species *i* in *j* and *k* plots, respectively.

The β_J_ is based on the incidence to measure the compositional dissimilarity between the plots based only on the differences in species occurrence (presence/absence), and it ranges from 0 (when both plots have the same species) to 1 (when the plots do not share any species; Catalán et al., 2023). Podani and Schmera (2011) proposed that the β_J_ consists of two components: the first component (Rrel = 2 min(b,c)/n) accounts for species replacement, and the second (Drel = |b − c|/n) accounts for richness differences. β_J_ was calculated using the following equation:


$$\begin{array}{l} \beta_{J}=\frac{b+c}{a+b+c}=\frac{number\;of\;species\;not\;shared}{total\;number\;of\;species}\\ \;=\frac{2min\left(b,c\right)}{a+b+c}+\frac{\left|b-c\right|}{a+b+c} \end{array}$$

#### 2.3.3 Phylogenetic structure

**Figure 2:**
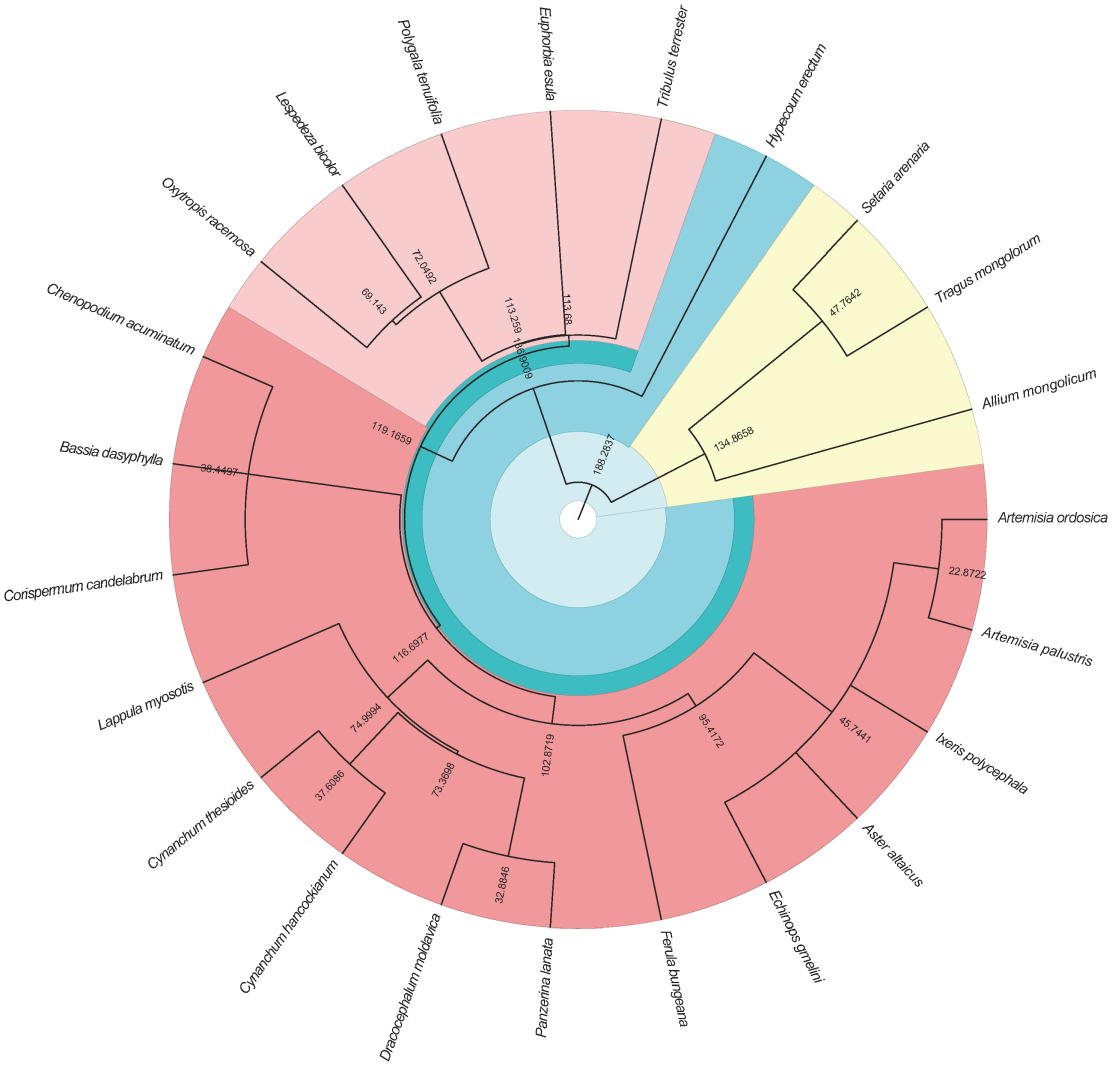
The phylogenetic tree of the plant species in the survey. The branch is the distance between the species.

Based on the list of species from the survey, the Chinese Flora (http://www.iplant.cn/frps) and the Plant List website (http://www.theplantlist.org/) were used to standardize the descriptions (Kalwij, 2012). To construct the phylogenetic tree, the awk version of the “phylomatic” software was used, and the phylogenetic tree was visualized using the “ggtree” package in R (Zheng et al., 2019b; **Figure 2**).

To characterize the phylogenetic diversity (PD), the PD index was used, which represents the sum of the lengths of the evolutionary branches of all the species in the community, and the phylogenetic structure was measured using the net relatedness index (NRI) and the net nearest taxon index (NTI). The PD index and phylogenetic structure were calculated using the “picante” package in R (Chai et al., 2016; Qian and Sandel, 2017).

The NRI and NTI were calculated using the following equations:


$$NRI=-1\times \frac{MPD_{s}-MPD_{mds}}{SD\left(MPD_{mds}\right)}$$

$$NTI=-1\times \frac{MNTD_{s}-MNTD_{r}}{SD\left(MNTD_{r}\right)}$$

where MPD is the mean phylogenetic distance between all the species pairs in the community, and MPD_s_ and MNTD_s_ denote the average observed paired phylogenetic distance and the average observed nearest neighbor phylogenetic distance, respectively. MPD_mds_ and MNTD_mds_ denote the average paired phylogenetic distance and the average nearest neighbor phylogenetic distance, respectively, under 999 null model simulations. SD is the standard deviation.

### 2.4 Data analysis

The statistical analyses were performed in the R platform (version 3.5.3). The significant differences between the different communities for the diversity indicators were evaluated using the Kruskal–Wallis rank sum test and the “dplyr” library. The overall change in the species composition was measured using a similarity percentage analysis (SIMPER) and the “vegan” package to calculate the contribution of the individual species to the overall community dissimilarity. The β-diversity was separated into two components, species replacement [or turnover (Repl)] and richness differences [or nestedness (RichDiff)], to clarify their relative contribution using the “adespatial” package. To examine whether the ST and NT communities were spatially similar, non-metric multidimensional scaling and hierarchical clustering were performed based on the Raup–Crick distance using the “vegan” library. Then, a correlation analysis was conducted between the phylogenetic and species diversity indices using the “corrplot” package. The roles of the deterministic and stochastic processes in community assembly were assessed using the β-null model (Tucker et al., 2016).

## 3 Results

### 3.1 Species composition and diversity

The survey revealed that there were 20 species of seed plants from 12 families and 20 genera identified in the ST communities, with Asteraceae having the most species (20.0%), followed by Amaranthaceae (15.0%). The proportion of the remaining families was less than 10.0%. There were nine herbaceous annuals, nine herbaceous perennials, and two semi-shrub species, with *Setaria arenaria* and *Corispermum candelabrum* (**Supplementary Table S1**). A total of 15 species of seed plants from eight families and 14 genera were identified in the NT communities, among which Asteraceae had the highest number of species (33.3%), which was followed by Amaranthaceae (20.0%) and Gramineae (13.3%), and the proportion of the remaining families was less than 10.0%. There were nine herbaceous annuals, four herbaceous perennials, one semi-shrub species, and one shrub species, with *A. ordosica* being the dominant species (**Supplementary Table S1**).

#### 3.1.1 α-diversity

The plant community in the shallow-tilled area had a high species diversity and evenness, with an H, D, J, and richness (S) of 1.68 ± 0.18, 0.72 ± 0.07, 0.66 ± 0.07, and 12.90 ± 2.85, respectively. In the control area, those measures were 0.98 ± 0.44, 0.46 ± 0.20, 0.54 ± 0.16, and 6.10 ± 2.38, respectively, and they were significantly higher (p < 0.05) in the shallow plowed area than in the control area (**Table 1**).

**Table 1:** Species diversity indices of the different communities.

Community type	ST	NT
Shannon–Weiner index (H)	1.68 ± 0.18a	0.98 ± 0.44b
Simpson index (D)	0.72 ± 0.07a	0.46 ± 0.20b
Pielou’s evenness index (J)	0.66 ± 0.07a	0.54 ± 0.16b
Richness (S)	12.90 ± 2.85a	6.10 ± 2.38b

Different lowercase letters in the same row indicate significant differences between the two data groups (p < 0.05).

ST, shallow tillage area; NT, non-shallow tillage area.

#### 3.1.2 β-diversity

**Figure 3:**
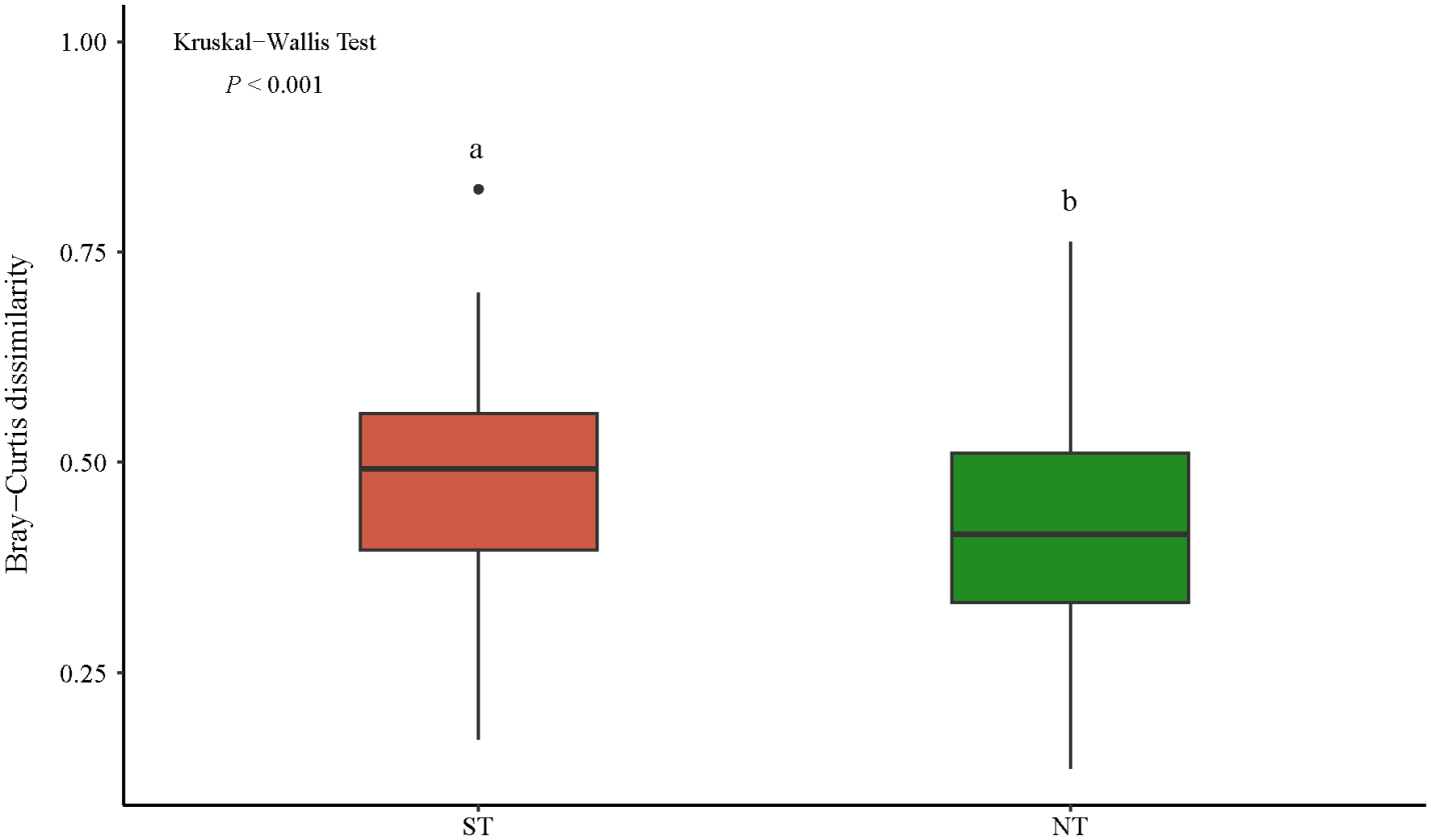
Differences in the Bray–Curtis dissimilarity index between the communities. The horizontal lines indicate the median values, the boxes indicate 25%–75% confidence intervals, the vertical lines indicate 10%–90% confidence intervals, the solid circles indicate outliers, and the different lowercase letters indicate significant differences among the communities. ST, shallow tillage area; NT, non-shallow tillage area.

**Figure 4:**
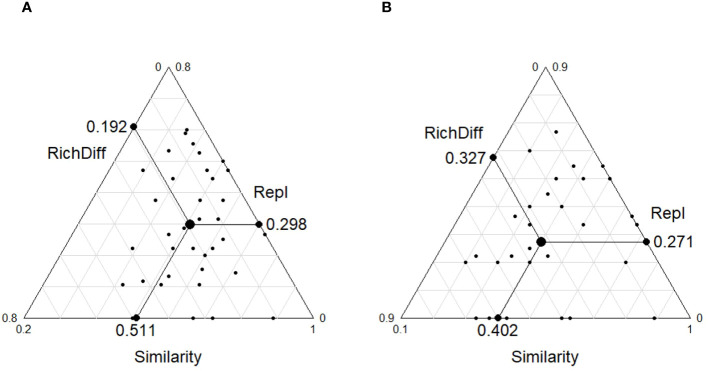
Triangular plots of the β-diversity comparisons (using the Jaccard index) for the plant communities among the **(A)** shallow tillage area (ST) and **(B)** non-shallow tillage area (NT). Each point represents a pair of sites. Its position is determined by a triplet of values, namely, the similarity (S), replacement (Repl), and richness difference (RichDiff) matrices, with each triplet summing to 1. The mean values of S, Repl, and RichDiff are shown.

The β-diversity analysis that was based on the Bray–Curtis dissimilarity index showed that the mean values of the β-diversity of the plant communities in the ST and NT communities were 0.48 and 0.42, respectively, and there was a highly significant difference between them (p < 0.001, **Figure 3**). The β-diversity decomposition analyses showed that the species replacement process contributed more (60.82%) to the differences in the plant community composition in the ST, and the richness differences contributed less to the β-diversity (**Figure 4A**). The differences in the richness and species turnover processes contributed similarly to the differences in the plant community composition in the NT, being 54.68% and 45.32%, respectively (**Figure 4B**).

The Venn diagram (**Supplementary Figure S1**) shows the presence of 13 common species between the two communities. Of the reported species in this survey, eight were exclusively identified in the ST (*Ferula bungeana*, *Oxytropis racemose*, *Polygala tenuifolia*, *Allium mongolicum*, *Dracocephalum moldavica*, *Tribulus terrestris*, *Panzeria alaschanica*, and *Cynanchum thesioides*), and three were only found in the NT (*Tragus mongolorum*, *Hypecoum erectum*, and *A. ordosica*).

The results of SIMPER analysis showed that *C. candelabrum*, *S. arenaria*, and *Chenopodium acuminatum* contributed the most to the inter-community species differences and that the contribution of the three plants to the inter-community dissimilarity between the ST and the NT was more than 10% (*C. candelabrum*, 18.44%; *S. arenaria*, 17.52%; *C. acuminatum*, 10.57%). The three plants’ cumulative contribution to the inter-community variation amounted to 68.13%, while the contribution of the other four plants to the inter-community differences amounted to 10.57%. The cumulative contribution of the seven plants to the inter-community variation amounted to 86.87% (**Table 2**).

**Table 2:** Contribution of the main species to community dissimilarity.

Species	Average contribution	Cumulative contribution
*Corispermum candelabrum*	0.1844	0.3348
*Setaria arenaria*	0.1752	0.5253
*Chenopodium acuminatum*	0.1057	0.6813
*Grubovia dasyphylla*	0.0749	0.7384
*Aster altaicus*	0.0355	0.7911
*Ixeris chinensis* subsp. *versicolor*	0.0235	0.8363
*Euphorbia esula*	0.0147	0.8687

### 3.2 Phylogenetic structure

**Figure 5:**
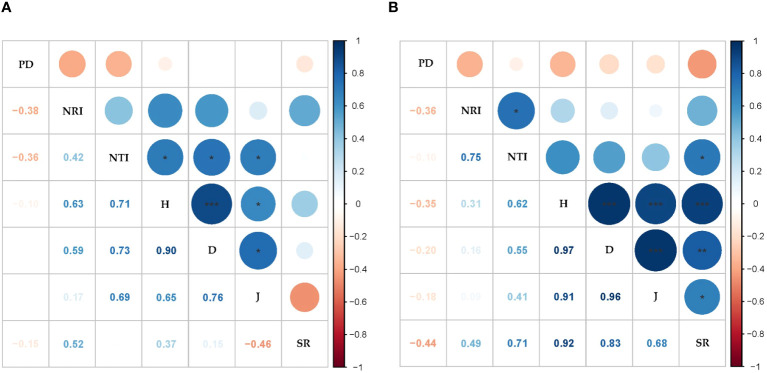
Correlation analysis between the species diversity and phylogenetic indices. **(A)** Shallow tillage area (ST). **(B)** Non-shallow tillage area (NT). Blue indicates a positive correlation, and orange indicates a negative correlation; the darker the color, the stronger the correlation. PD, phylogenetic diversity; NRI, net relatedness index; NTI, net nearest taxon index; H, Shannon–Weiner index; D, Simpson’s index; J, Pielou’s evenness index; SR, species richness. *p < 0.05; **p < 0.01; ***p < 0.001.

The mean values of the PD, NRI, and NTI were 1,251.06 ± 70.94, −0.74 ± 0.03, and −0.06 ± 0.18 in the ST, respectively; the mean values of the PD, NRI, and NTI were 683.26 ± 62.37, −0.89 ± 0.13, and −0.84 ± 0.51 in the NT, respectively. The PD was significantly higher in the ST than in the NT, and the NRI was significantly lower in the ST than in the NT (p < 0.001, **Supplementary Table S2**). No significant correlation was found between the PD values and species diversity indices in the ST and NT communities. In the ST communities, the NTI was significantly and positively correlated with the H, D, and J (p < 0.001; **Figure 5A**). The NTI and NRI were significantly and positively correlated with the SR in the NT communities (p < 0.05, **Figure 5B**).

### 3.3 Community assembly process

**Figure 6:**
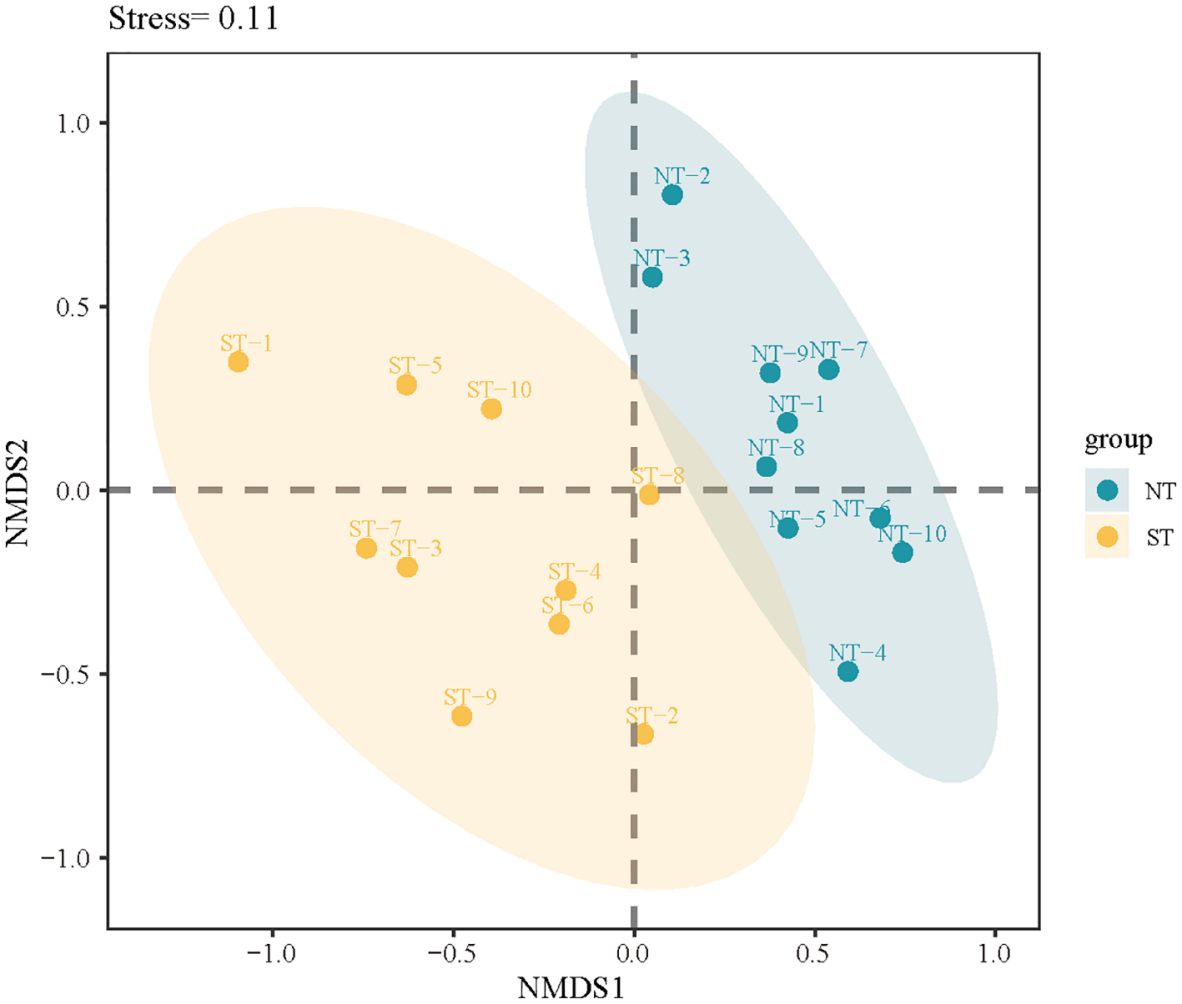
Non-metric multidimensional scaling (NMDS) analysis of the species composition in the sample plots. ST, shallow tillage area (yellow circle); NT, non-shallow tillage area (blue circle).

The results revealed that the NT communities were more similar, indicating that they deviated from the null hypothesis (**Figure 6**). However, the ST communities were farther apart and spread over half of the space, suggesting that the null hypothesis may be supported. These results suggest that stochastic processes play a greater role in the ST communities. A similar result was observed for the phylogenetic structure. In the ST and NT communities, the NRI and NTI were both less than 0 (**Supplementary Table S2**), indicating that the phylogenetic structure of the plant community diverged, there were more distantly related species in the community, the construction of the ST and NT communities was dominated by stochastic processes, and competitive exclusion was the primary mechanism of plant construction. However, when compared with the NT communities, the NRI and NTI were closer to 0 in the ST communities, indicating that the ST communities were subject to stronger stochastic processes.

## 4 Discussion

### 4.1 Species diversity and species composition

The plant community structure and function are determined by a variety of biotic and abiotic drivers, but anthropogenic environmental changes may alter these drivers and their effects on the plant communities (Brooker, 2006). The Mu Us Desert, as a typical agricultural and pastoral area, is ecologically fragile and frequently disturbed by human activities, and a full understanding of the biodiversity changes before and after disturbance is needed for effective conservation. Studies have confirmed that the SR of woody and herbaceous plants is strongly affected by environmental changes and anthropogenic disturbances (Zhou et al., 2018). Shallow tillage removes almost all the existing above-ground vegetation, increases the ecological niche space for communities (Tortorelli et al., 2022), and alters the physical properties, such as soil compactness, creating open and variable soil conditions and promoting soil seed germination and organic matter decomposition (Lu et al., 2018). Herbaceous plants, whose seeds are generally small and easy to disseminate, can quickly and effectively utilize temporary habitats for reproduction and respond to external disturbances than woody plants, which resulted in a significantly higher species diversity index in the ST communities than in the NT communities. Additionally, the allelopathic effect of *A. ordosica* significantly inhibits seed germination and seedling growth of herbaceous plants (Lopes et al., 2022). When a layer of up to 80% shrubs is formed, annual and biennial herbaceous plants are disadvantaged in resource competition and are mostly distributed in the unshaded edges of *A. ordosica* scrub. Thus, competition for available resources, such as light and soil nutrients, influences the maintenance of species diversity (Adler et al., 2018). At the same time, the stochastic nature of seed dispersal produced a preferential effect (Weidlich et al., 2021), with large differences in the species composition among the ST communities, which were clearly differentiated from the NT communities. Analyses of the β-diversity also revealed differences in the species composition between the ST and NT communities, with the β_ST_ being dominated by species replacement processes, indicating that the different communities tended to have more endemic species, and differences in abundance played a larger role than species replacement processes in the β_NT_. This showed that a few communities contained the vast majority of the species in that state.

Several studies have demonstrated a negative correlation between the community restoration time and the SR; with the extension of the restoration time, early colonizing herbaceous plants will largely disappear (Daws et al., 2021; Standish et al., 2021). Ecological communities are always dynamic and vary in space and time, and they often develop along relatively predictable successional trajectories (Mori et al., 2018). We speculate that without re-imposed anthropogenic disturbance, the ST community will become more similar to the NT and form a zonal sub-top-level community with *A. ordosica* as a single dominant species (Webb et al., 1987; Li et al., 2015), i.e., a convergent process of community succession.

### 4.2 Phylogenetic structure

The low PD values shown for the NT communities suggested that fewer clades successfully colonized the NT and that the NT has a high frequency of closely related species. This could reflect the fact that phylogenetic diversity is lower in environments with greater competitive pressures for resources, as these environments may represent ecophysiological barriers that are difficult to surmount evolutionarily (Honorio Coronado et al., 2015); however, due to the small sample size and the limited extent of plots, further research is needed.

The increase in the SR may imply more complex interspecific affinities, which in turn leads to increased PD values, but no significant positive correlation between the PD values and the species diversity index was found in both the ST and NT in this study. Most studies have demonstrated a correlation between the two, but the correlation decreases with unbalanced evolutionary trees or narrow species distributions (Gascuel et al., 2015). When the ST and NT communities were viewed as a whole, the PD, NTI, and NRI were significantly positively correlated with the H, D, and S (**Supplementary Figure S2**). This significant relationship indicates that the species composition and distribution have some influence on the phylogenetic structure.

### 4.3 Community assembly

The phylogenetic structure of both the ST and NT communities tended to diverge (NTI < 0 and NRI < 0), suggesting that competitive exclusion is a dominant mechanism influencing community assembly. This pattern may be attributed to the limited availability of soil water and nutrients in the Mu Us Desert, which likely prevents closely related species with similar ecological niches from coexisting due to competition for these scarce resources, ultimately leading to the strengthening of interspecific ecological niche differentiation and the dispersion of phylogenetic structure (Webb et al., 2002; Silvertown, 2004). It is important to note that shallow tillage did not alter the major ecological processes driving community assembly, but the relative importance of deterministic and stochastic processes differed between the two treatments. The NRI and NTI of the ST communities differed significantly from those of the NT communities (p < 0.05, **Supplementary Table S2**), and analysis based on the Raup–Crick dissimilarity index also showed that the β_RC_ within the ST communities was closer to the zero expectation than in the NT communities (**Supplementary Figure S4**), suggesting that in addition to being dominated by competitive exclusion, the ST was also largely influenced by stochastic processes dominated by dispersal limitation, which resulted in lower species similarity within the communities. Studies have confirmed that disturbance may promote stochastic processes (Didham et al., 2005; Didham and Norton, 2006).

The desert maintains environmental consistency at large scales (Teramoto et al., 2022), and it can be assumed that the effective seed bank is the result of deterministic abiotic factors, such as temperature or precipitation, and stochastic factors, such as dispersal constraints. In contrast, habitat sieving at small scales mainly consists of biotic factors, such as interspecific interactions (Royo and Ristau, 2013; Zou et al., 2021). As *A. ordosica* requires a large amount of resources as a community-building species, including light, it results in a limited number of ecological niches being available in the community, which prevents certain species from persisting (Deng et al., 2022). In contrast, under shallow tillage conditions, where the community species are essentially removed and available ecological niches proliferate, herbaceous seeds can germinate rapidly and complete their life history cycles when the climatic conditions are favorable (Grime, 1977). Under these conditions, rapid reproductive processes may reduce the role of severe constraints on the hydrothermal conditions and inter- and intraspecific competition in shaping community membership. In future investigations, more attention should be paid to the role of annual and biennial herbaceous plants in the process of community assembly under human interference.

## 5 Conclusions

Shallow tillage increases species alpha, beta, and phylogenetic diversity and reduces the importance of competitive exclusion in community assembly. Therefore, we suggest that plant species with low ecological niche overlap should be selected for artificial vegetation restoration in the Mu Us Desert to reduce competition for the same resources.

## Data availability statement

The original contributions presented in the study are included in the article/**Supplementary Material**. Further inquiries can be directed to the corresponding authors.

## Author contributions

ZL: Conceptualization, Investigation, Data curation, Writing – original draft, Writing – review & editing. JQ: Writing – original draft, Writing – review & editing. ZL: Investigation, Writing – review & editing. XG: Investigation, Writing – review & editing. GH: Investigation, Writing – review & editing. HY: Investigation, Writing – review & editing. EH: Investigation, Writing – review & editing. CL: Investigation, Writing – review & editing. XW: Writing – review & editing. GL: Writing – review & editing, Funding acquisition. RG: Writing – review & editing, Supervision.
