# Supplementary material for: Shallow tillage mitigates plant competition by increasing diversity and altering plant community assembly process
Source: Front Plant Sci. 2024 Aug 7;15:1409493. doi: 10.3389/fpls.2024.1409493 (PMC11335505; doi:10.3389/fpls.2024.1409493)
Supplement: Supplementary file 1 [file DataSheet_1.docx]

Supplementary Material

# Supplementary Tables

**Supplementary Table 1**. Species presence/absence data within the different community samples. 1 indicates presence, 0 indicates absence. ST, shallow tillage; NT, non-shallow tillage.

| Name of the plant | Species presence/absence matrix | |
| --- | --- | --- |
|  | shallow tillage (ST) communities | non-shallow tillage (NT) communities |
| *Vincetoxicum mongolicum* Maxim. | 1 | 1 |
| *Lespedeza bicolor* Turcz. | 1 | 1 |
| *Lappula myosotis* Moench | 1 | 1 |
| *Grubovia dasyphylla* (Fisch. & C. A. Mey.) Freitag & G. Kadereit | 1 | 1 |
| *Ferula bungeana* Kitag. | 1 | 0 |
| *Oxytropis racemosa* Turcz. | 1 | 0 |
| *Polygala tenuifolia* Willd. | 1 | 0 |
| *Echinops gmelinii* Turcz. | 1 | 1 |
| *Ixeris chinensis* (Thunb.) Nakai | 1 | 1 |
| *Setaria arenaria* Kitag. | 1 | 1 |
| *Allium mongolicum* Regel | 1 | 0 |
| *Corispermum candelabrum* Iljin | 1 | 1 |
| *Dracocephalum moldavica* L. | 1 | 0 |
| *Tribulus terrestris* L. | 1 | 0 |
| *Cynanchum thesioides* (Freyn) K. Schum. | 1 | 0 |
| *Artemisia palustris* L. | 1 | 1 |
| *Aster altaicus* Willd. | 1 | 1 |
| *Chenopodium acuminatum* Willd. | 1 | 1 |
| *Panzerina lanata* var. *alaschanica* (Kuprian.) H. W. Li | 1 | 0 |
| *Euphorbia esula* L. | 1 | 1 |
| *Hypecoum erectum* L. | 0 | 1 |
| *Tragus mongolorum* Ohwi | 0 | 1 |
| *Artemisia ordosica* | 0 | 1 |

**Supplementary Table 2**. Species diversity index of the different communities. ST, shallow tillage; NT, non-shallow tillage. PD: phylogenetic diversity; NRI: net relatedness index; NTI: net nearest taxon index. The different lowercase letters in the same row indicate significant differences between the two data groups (P < 0.001).

| Community type | ST | NT |
| --- | --- | --- |
| PD | 1251.06±70.94a | 683.26±62.37b |
| NRI | -0.74±0.03b | -0.89±0.17a |
| NTI | -0.06±0.18a | -0.84±0.51b |

# Supplementary Figures


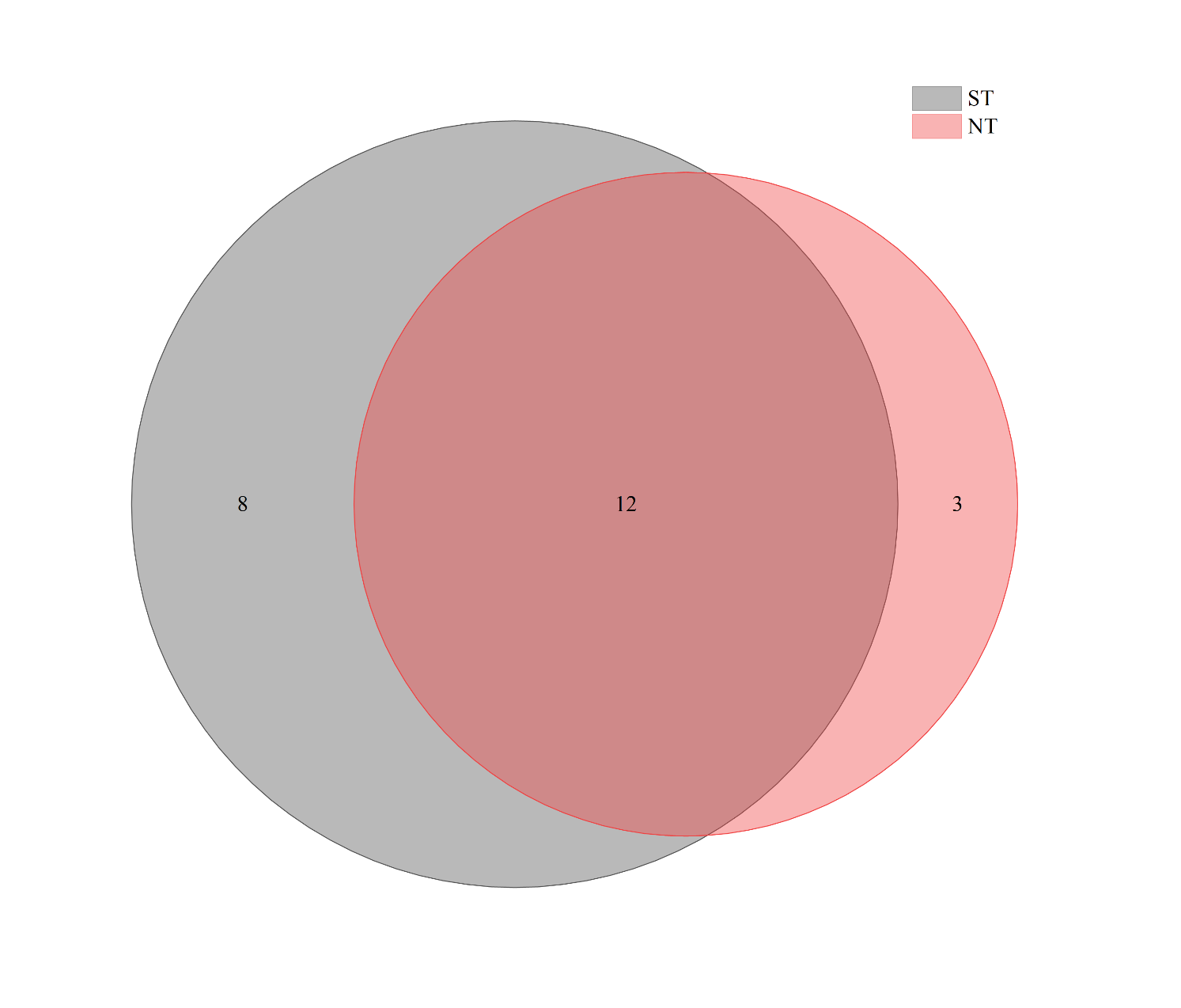


**Supplementary Figure 1.** Venn diagram showing the similarities and differences between the different communities. ST, shallow tillage; NT, non-shallow tillage.


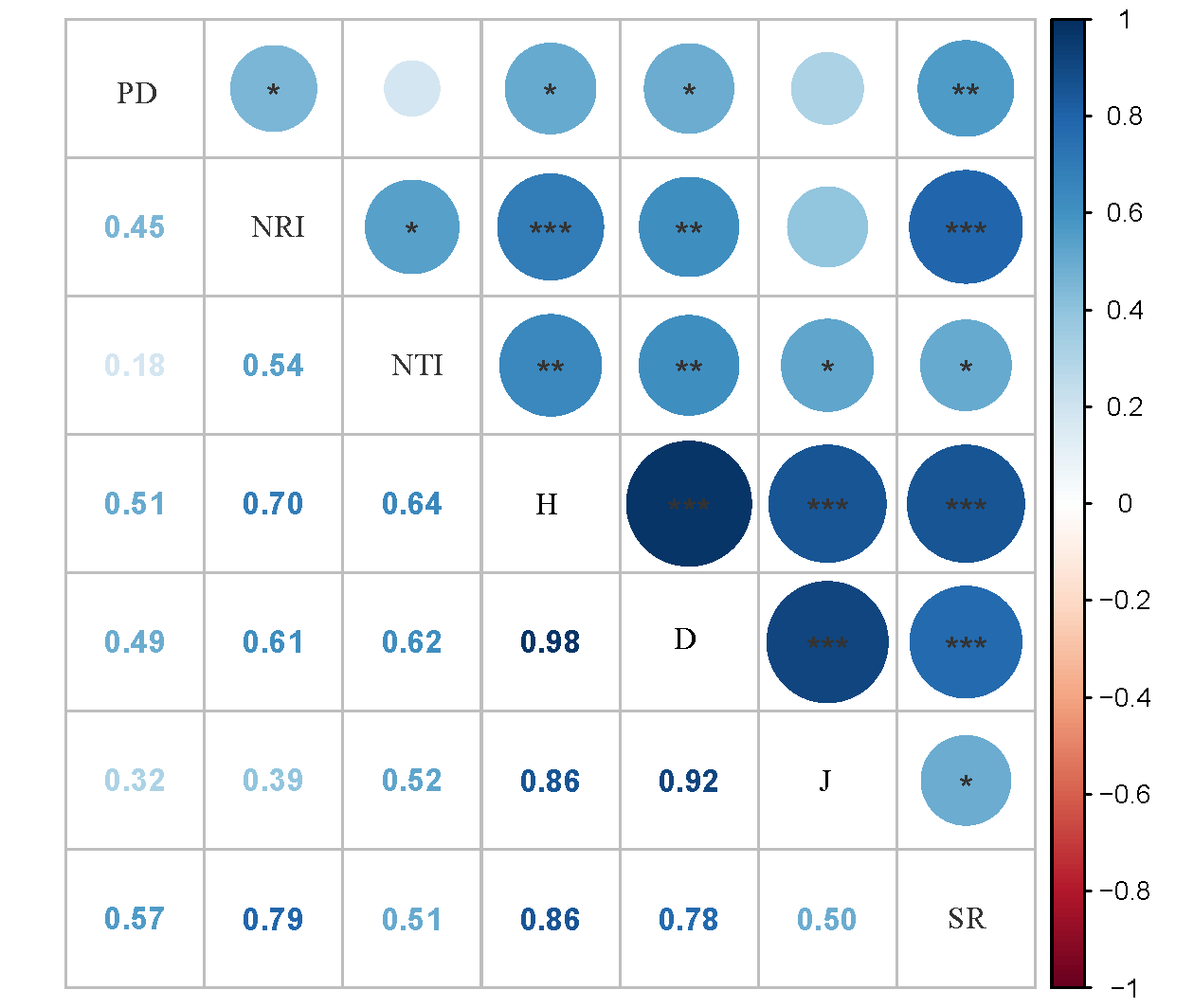


**Supplementary Figure 2.** Correlation between the species diversity indices and phylogenetic structure indices after considering the ST and NT communities as a whole. PD: phylogenetic diversity; NRI: net relatedness index; NTI: net nearest taxon index; H: Shannon-Wiener index; D: Simpson's index; J: Pielou evenness index; SR: species richness; *: (P < 0.05); **: (P < 0.01); ***: (P < 0.001).


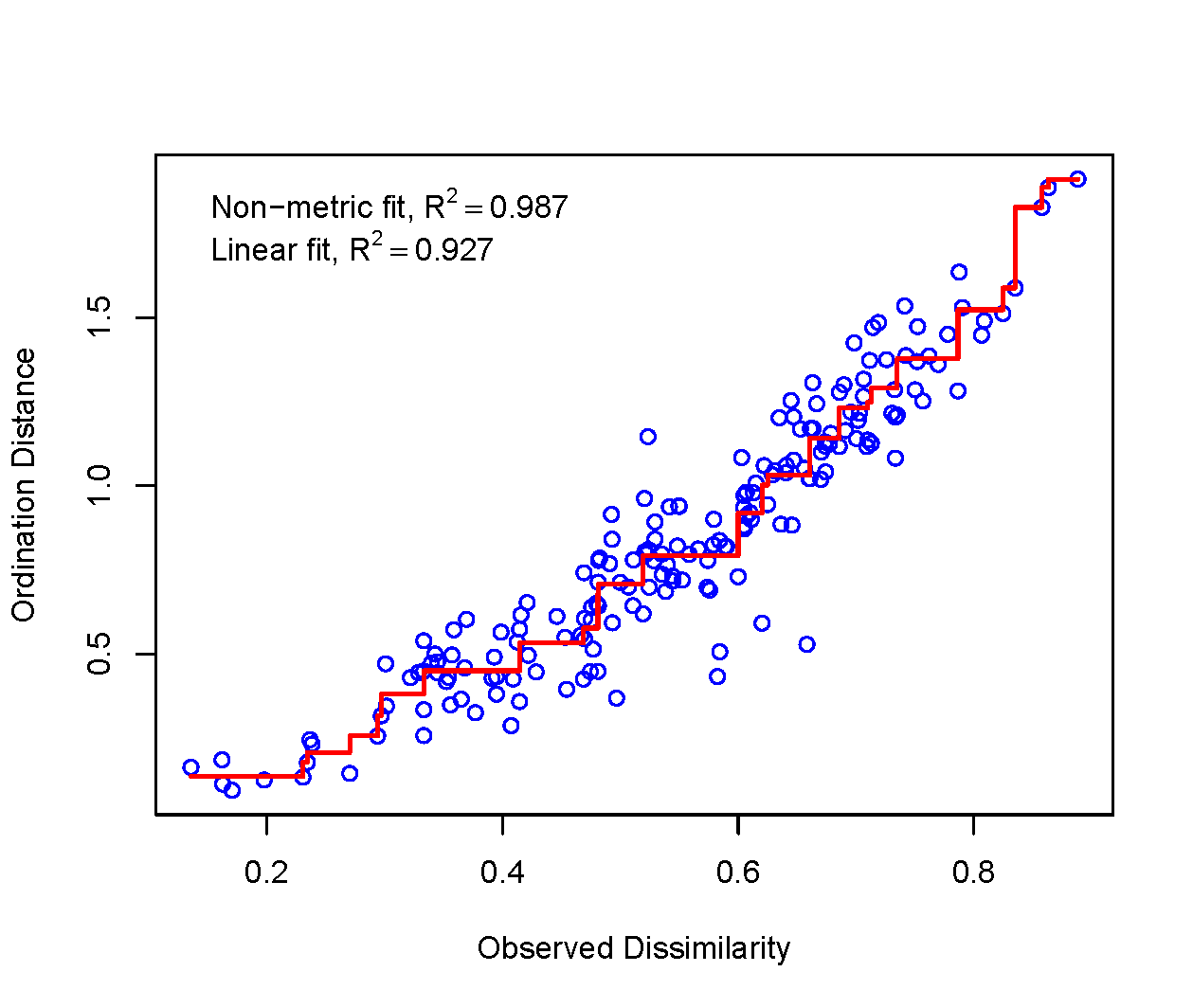


**Supplementary Figure 3.** A check of the relationship between the non-similarity of observations and the sorting distance. The absence of points distributed farther down the line indicates that this data can be analyzed using NMDS.


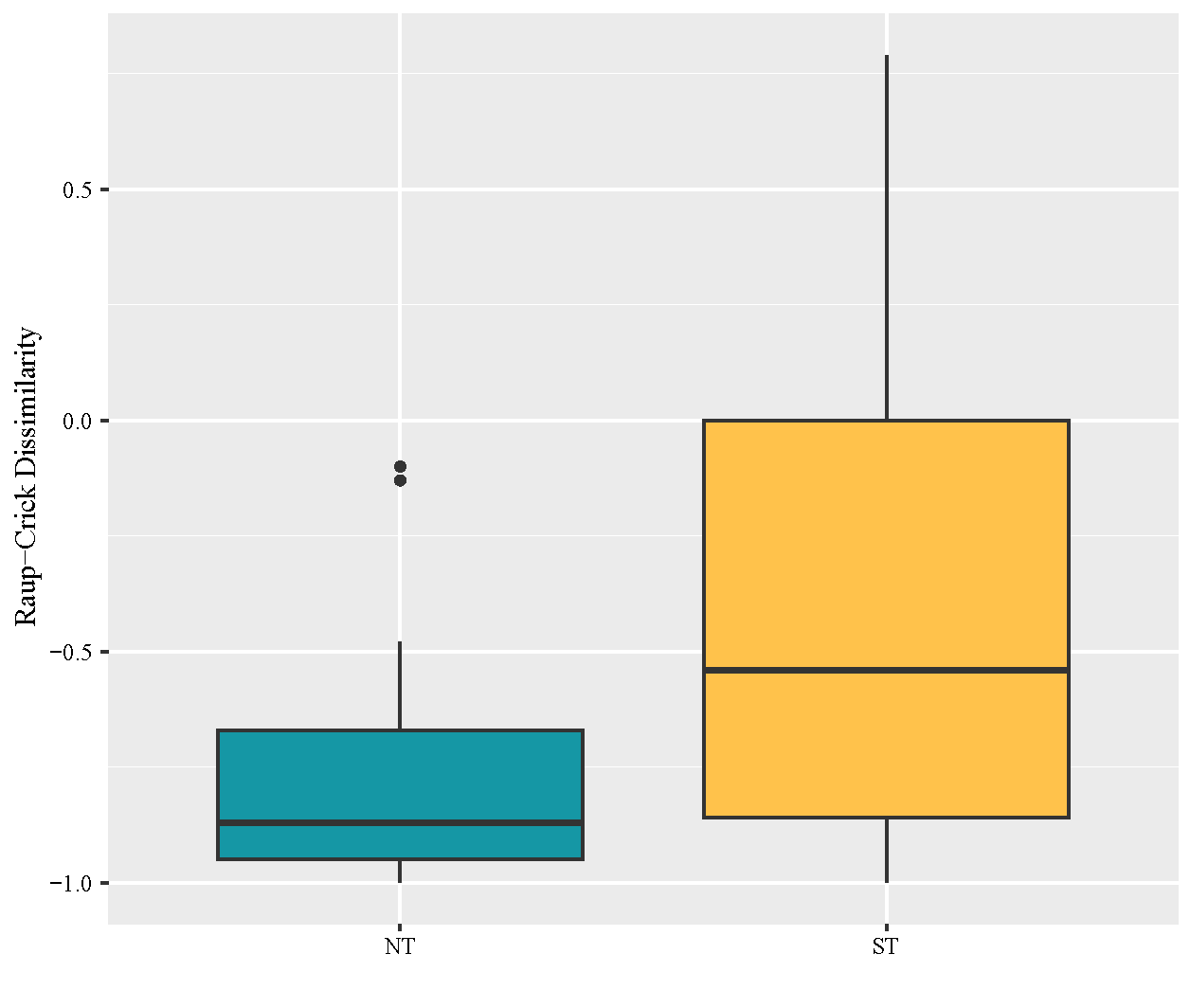


**Supplementary Figure 4.** Raup-Crick dissimilarity index analyses, applied to Beta diversity analyses, indicate the degree of similarity (or dissimilarity) between two communities, and also provide some indication of the possible underlying mechanisms of community assembly. A value of 0 indicates that the observed degree of similarity (or dissimilarity) is indistinguishable from the null expectation, and a value of 1 indicates that the observed difference is higher than the null expectation of any simulation (the difference between communities is entirely greater than what would be expected by chance), and vice versa. expectation), and -1 indicates that the observed difference is below the zero expectation of any simulation (the difference between communities is entirely less than what would be expected by chance). ST, shallow tillage; NT, non-shallow tillage.
